
JOURNAL INFORMATION
==============================
NLM Title Abbreviation: ScientificWorldJournal
Journal ID: ScientificWorldJournal
NLM Journal ID: 101131163
Journal ID: TSWJ

The Scientific World Journal

ISSN: 2356-6140
EISSN: 1537-744X
Publisher: Wiley

ARTICLE INFORMATION
==============================
PMCID: PMC6084505
PMID: 30147498
DOI: 10.1100/tsw.2001.150
Article ID: 605065
Article version: 1
Subjects: Short Report

Mapk/Sapk Signaling in Apoptosis and Bfgf-Mediated Survival

Schamberger Chantal *
Cerni Christa
Institute of Cancer Research, University of Vienna, A-1090, Austria
*Corresponding author.
Publication date: 2001
Electronic publication date: 2001 Nov 30
Volume: 1
First page: 43
Last page: 43
Copyright: Copyright © 2001 Chantal Schamberger and Christa Cerni.
Copyright year: 2001
License: This is an open access article distributed under the Creative Commons Attribution License, which permits unrestricted use, distribution, and reproduction in any medium, provided the original work is properly cited.
License URL: https://creativecommons.org/licenses/by/3.0/

==============================
Miami *Nature Biotechnology* Short Reports
*TheScientificWorld* (2001) 1(S3), 43SR
ISSN 1532-2246; DOI 10.1100/tsw.2001.150

### MAPK/SAPK SIGNALING IN APOPTOSIS AND BFGF-MEDIATED SURVIVAL

Chantal Schamberger* and Christa Cerni

Institute of Cancer Research, University of Vienna, A-1090, Austria
* Corresponding author.

**INTRODUCTION.** MAPK/SAPK signalling cascades play an important role in proliferation, differentiation, cytokine production and cytoskeletal organization, depending on stimuli and cell type (1). These pathways are also involved in other important cellular processes such as apoptosis and survival, although these mechanisms are less understood. The embryonic rat cell line 423 undergoes prompt apoptosis upon serum withdrawal, which can be prevented by addition of basic fibroblast growth factor (bFGF). Since c-Ha-ras-supertransfected 423 cell clones also resisted serum reduction, we studied the involvement of MAPK/SAPK signaling pathways during apoptosis and bFGF-mediated rescue (2).

**METHODS.** 423-cells were cultivated with or without serum and apoptotic cells were analyzed by CASY™ Cell Counter System (determination of cell number and cell diameter) and by Western blot analysis (e.g. Casp-3, p38, Erk1/2). Two MEK1 inhibitors (PD98059 and U0126; Calbiochem) and three p38/SAPK2 inhibitors (SB202190, SB203580 and PD169513; Calbiochem) were applied.

**RESULTS.** Upon serum withdrawal detachment of 423 cells started after three hours and, by eight hours, approximately 60% of the cells were apoptotic. This was accompanied by chromatin condensation, DNA-laddering and cleavage of casp-3. Addition of bFGF to serum-deprived confluent cells efficiently prevented detachment of cells and DNA fragmentation. During apoptosis phosphorylation of Erk1/2 started to decline after half an hour upon serum withdrawal and was reactivated after 12 hours, when the majority of cells had apoptosed (Fig.1). Upon addition of bFGF dephosphorylation of Erk1/2 was considerably retarded compared to apoptotic conditions. During apoptosis, phosphorylation of p38/SAPK2 was strongly, but transiently induced three hours after serum withdrawal. After 24 hours, when the majority of cells had detached and apoptosed, p38/ SAPK2 was found again activated in the remaining viable spare cell layer (Fig.2). Addition of PD98059 [25µM] and U0126 [10µM] inhibited Erk1/2 phosphorylation and slightly enhanced apoptosis (3), while bFGF-mediated rescue seemed to be independent of MEK1 activation. Application of three different p38/SAPK2 inhibitors [25µM each] exerted strikingly distinct effects. SB202190 and SB 203580 induced proliferation of 423 cells, but had no effect on apoptosis. In contrast PD169513 inhibited apoptosis, but did not stimulate proliferation. None of the tested p38/SAPK2 inhibitors was able to abrogate bFGF-mediated survival.

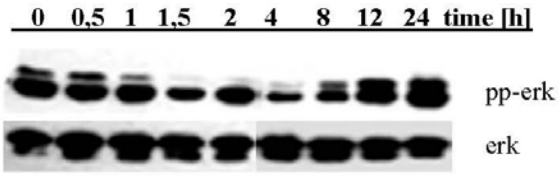

Fig.1 Erk1/2 phosphorylation during apoptosis

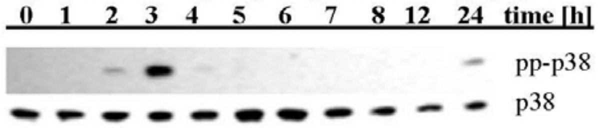

Fig.2 p38/SAPK2 phosphorylation during apoptosis

**DISCUSSION.** The contribution of MAPK/SAPK signaling during apoptosis is non-uniform and depends on stimuli and cell type (4). Two different MEK1 inhibitors altered neither the kinetics of apoptosis, nor bFGF-mediated survival, indicating that this pathway was not involved in either cellular reaction. Surprisingly, addition of PD169513 prevented apoptosis in a concentration dependent manner, while two other p38/SAPK2 inhibitors, SB202180 and SB 203590, exerted little or no effects on apoptosis and survival. This suggests that the inhibitors affect different isoforms of p38. We are currently studying which of them is relevant for apoptotic signalling by means of immunoprecipitation and kinase assays of flag-tagged-p38 isoforms. Since bFGF induced survival irrespective of MEK1- and p38- inhibition, we assume that bFGF acts either further downstream in these cascades or by an independent route.

**REFERENCES.**

1. Maher, P.A. (1999) J. Biol. Chem. 274, 17491
2. Terada, N., Katiro, Y., and Satoh, T. (2000) Biochem. Biophys. Res. Commun. 267, 449
3. Le Gall, M., Chambard, J.-C., et al. (2000) Mol. Biol. Cell 11, 1103
4. Ono, H. and Han, J. (2000) Cell. Signal. 12, 1

This work was supported by the Jubilaeumsfond der Oesterreichischen National Bank.
